# Neodymium Recovery from the Aqueous Phase Using a Residual Material from Saccharified Banana-Rachis/Polyethylene-Glycol

**DOI:** 10.3390/polym15071666

**Published:** 2023-03-27

**Authors:** Byron Lapo, Sandra Pavón, Martin Bertau, Hary Demey, Miguel Meneses, Ana María Sastre

**Affiliations:** 1 Department of Chemical Engineering, Universitat Politècnica de Catalunya, ETSEIB, Diagonal 647, 08028 Barcelona, Spainana.maria.sastre@upc.edu (A.M.S.); 2 School of Chemical Engineering, Universidad Técnica de Machala, UACQS, BIOeng, Machala 070151, Ecuador; 3 Institute of Chemical Technology, TU Bergakademie Freiberg, Leipziger Straße 29, 09599 Freiberg, Germany; sandra.pavon.regana@ikts.fraunhofer.de (S.P.); martin.bertau@chemie.tu-freiberg.de (M.B.); 4 Fraunhofer Institute for Ceramic Technologies and Systems IKTS, Fraunhofer Technology Center for High-Performance Materials THM, Am St.-Nicklas-Schacht 13, 09599 Freiberg, Germany; 5 Department of Chemistry, Universidad Técnica Particular de Loja, San Cayetano Alto, Loja 110150, Ecuador; mameneses@utpl.edu.ec

**Keywords:** rare earth recovery, sorption, lignocellulosic waste, PEG

## Abstract

Neodymium (Nd) is a key rare earth element (REE) needed for the future of incoming technologies including road transport and power generation. Hereby, a sustainable adsorbent material for recovering Nd from the aqueous phase using a residue from the saccharification process is presented. Banana rachis (BR) was treated with cellulases and polyethylene glycol (PEG) to produce fermentable sugars prior to applying the final residue (BR–PEG) as an adsorbent material. BR–PEG was characterized by scanning electron microscopy (SEM), compositional analysis, pH of zero charge (pH_pzc_), Fourier transform infrared analysis (FTIR) and thermogravimetric analysis (TGA). A surface response experimental design was used for obtaining the optimized adsorption conditions in terms of the pH of the aqueous phase and the particle size. With the optimal conditions, equilibrium isotherms, kinetics and adsorption–desorption cycles were performed. The optimal pH and particle size were 4.5 and 209.19 μm, respectively. BR–PEG presented equilibrium kinetics after 20 min and maximum adsorption capacities of 44.11 mg/g. In terms of reusage, BR–PEG can be efficiently reused for five adsorption–desorption cycles. BR–PEG was demonstrated to be a low-cost bioresourced alternative for recovering Nd by adsorption.

## 1. Introduction

The recovery of REE, and particularly the Nd element, has gained attention due to the growing demand for the production of technological Nd-containing products. For example, the Nd element is part of permanent NdFeB magnets used in wind turbines, vehicles, mobile phones, CoSm magnets, hard disk drives, computers and peripherals [1]. The Nd element is considered a key element in the green energy technology market [2], particularly in road mobility and energy generation [3]. Over the last 30 years, a total of 880 kt of Nd was extracted from mines, 64% of which has not been recycled [4]. Accordingly, Nd was classified as a critical REE by the U.S. Department of Energy [5], which involves several actions, including promoting the development of technology in recycling areas to minimize the future provisioning issues. On the other hand, Nd has been recognized as a new contaminant in water resources [6]. Thus, the recovery of Nd is needed to ensure the future global demand and to minimize the environmental affectation caused by the discarding of technological devices.

Several methods have been developed for the recovery of Nd^3+^ ions from the aqueous phase such as solvent extraction, ion-exchange, co-precipitation and adsorption [7,8]. Among these technologies, adsorption and, particularly, biosorption are sustainable methods for recovering this element due to the use of bio-based materials. Lignocellulosic resources have gained attention in recent years because these biosorbent materials have demonstrated the capability for removing metals, dyes, REE and other contaminants [9,10,11]. Banana rachis (BR) is a lignocellulosic waste material which is able to recover various REE ions by the absorption of what is owed to the affinity of its carboxylic groups to REE [12]. Banana cultivation is of considerable economic importance; about 130 countries produce and export this fruit. The production of one metric ton of fruit generates four metric tons of wastes, which means that the production of approximately 115 million metric tons of fruit per year produces around 460 million metric tons of waste, including rachis, pseudo-stem and peel [13,14]. This means BR is not only an interesting and promising source for bio-sorbent material fabrication, but it also holds great potential for alleviating both waste problems and rare earth supply problems.

A now-established approach to valorizing lignocellulosic wastes is producing second-generation ethanol [15], but the solid residue after saccharification could be used as adsorbent material. The solid remnants mainly consist of residues of cellulose, hemicelluloses and lignin [16], which conserves some functional surface groups that are able to attach metals by the adsorption process.

Ethanol production from banana waste would start with converting cellulose into glucose as a fermentable sugar. Several approaches have been tested, including biological, physical-mechanical, chemical approaches or their combinations [17,18]. Polyethylene glycol (PEG) is a non-toxic, non-ionic surfactant for which there exists experience in enhancing saccharification during enzymatic hydrolysis (EH) [19]. Due to the amphiphilic nature of PEG, this surfactant can be adsorbed onto the lignocellulosic surface, facilitating the immobilization of enzymes on the substrate and avoiding the inhibition effect caused by the adsorption of cellulase on lignin and cellulose [20]. Alternatively, regarding metal adsorption, PEG has been successfully applied to improve the adsorption performance of acid orange II dye by the reduction in the agglomeration of the sorbent material particles, therefore allowing for the availability of more active surface sites [21]. The suitability of PEG for promoting both enzymatic lignocellulose digestion and metal binding prompted us to investigate the use of PEG in combining the capability of improving the saccharification process and the solid residue for adsorption purposes.

In fact, to the best of our knowledge, PEG has not been tested for improving the sugar conversion of BR, nor for producing sorbent materials with enhanced adsorbent properties from BR waste material. Here, the example of Nd^3+^ is a representative of the rare earth metal family. In this context, the current research focuses on evaluating the residue from enzymatic hydrolysis with PEG as an adsorbent material of Nd^3+^ ions from the aqueous phase. The evaluations include the parameters that affect the adsorption performance, such as the pH, particle size, initial Nd concentration and reaction time. We also tested to what extent the material can be reused. The present research contributes to the green chemistry concept through the incorporation of a renewable raw material based on a residue from a residue, seeking the avoidance of wastes and the use of degradable chemical products. This approach tends to improve the value chain and circular economy of the banana market, which, in some developing countries, is just limited to the exportation of banana fruit. 

## 2. Materials and Methods

### 2.1. Chemicals

Neodymium (III) nitrate hexahydrate (Nd(NO_3_)_3_·6H_2_O, 99.9%), sodium hydroxide (NaOH, 98%) and nitric acid (HNO_3_, 70%) were obtained from Alfa Aesar Inc. (Kandel, Germany). Polyethylene glycol 4000 (PEG), cellulase (enzyme blend of cellulases, ß-glucosidases and hemicellulose, Lot #SLBW7460) and citric acid (99.5%) were the products of Sigma Aldrich Inc. (St. Louis, MO, USA). Deionized water was used to prepare the solutions.

### 2.2. Adsorbent Material Preparation

The solid residue from the EH of BR treated with PEG was labeled as BR–PEG. This BR–PEG material was used as the adsorbent material. Prior to obtaining BR–PEG, the BR was collected from the Musa Cavendish plantation located in the south of Ecuador (3°15′ S, 80°51′ W). The neat BR was washed, chopped in pieces of around 2–5 cm, dried at 75 °C, milled and sieved. These particles were dried afterwards at 75 °C for 48 h. 

The batch EH experiments were performed in a 1 L reactor (Tecnal, Piracicaba, Brazil). The EH consisted of adding 50 g of BR in 1 L of distilled water and autoclaving at 121 °C for 15 min. The solid residue was transferred to a citrate buffer solution of pH 4.8. Then, 1 g of PEG (2% *w*/*w*) was added and kept in the blend for 1 h at room temperature (25 ± 2 °C). Finally, 3.25 mL of the cellulase blend was added to the reactor (30 FPU/g—enzymatic activity of 206.58 FPU/mL). The hydrolysis experiments were performed by keeping the temperature at 50 °C at 150 rpm for 96 h. The obtained solid residue (BR–PEG) was dried at 75 °C, milled and sieved to three particle size fractions: (i) 150–180 µm, (ii) 500–600 µm and (iii) 850–1000 µm. To analyze the data using Design-Expert 11 software, the averages of these particle size values were used, according to Table 1. The experiments were conducted in triplicate.

### 2.3. Material Characterization

The composition in terms of the quantity of cellulose, hemicellulose and lignin was analyzed by the ASTM D-1103-60, ASTM D-1104-56 and TAPPI 222 om-02 standard methodologies, respectively [22].

The pH point of zero charge (pH_pzc_) was determined by measuring the pH change in electrolyte solutions loaded with BR–PEG. A total of 25 mg of BR–PEG was added in 25 mL of the electrolyte solutions (adsorbent dosage: 1 g/L) of NaCl 0.01 M with an initial pH (pH_o_) of 3.0, 5.0, 7.0 and 9.0. These analyses were executed in triplicate.

A scanning electron microscope (SEM) coupled with an energy dispersive X-ray probe (EDX) was used to observe the morphology of the BR–PEG (Phenom XL, Eindhoven, The Netherlands). Prior to the observations, the BR–PEG was sputtered with a carbon film.

Fourier transform infrared (FTIR-ATR) spectroscopy was used to determine the surface functional groups (Spectrum Two IR, Perkin Elmer, Waltham, MA, USA).

Thermogravimetric analysis (TGA) was carried out from 25 to 800 °C, applying a heating rate of 10 K/min under a nitrogen atmosphere at a flow rate of 30 mL/min (TGA/DSC 3+, Mettler Toledo, Greifensee, Switzerland).

### 2.4. Adsorption Experiments

#### 2.4.1. pH and Particle Size Influence

To establish the optimal pH of the aqueous phase and the optimal particle size of BR–PEG to recover Nd^3+^ ions, a response surface methodology (RSM) based on central composite design (CCD) with two factors and face-centered was conducted. The experiments were run in duplicate, and the central point was replicated five times. Table 1 shows the factors and levels evaluated.

The adsorption capacity (*q_e_*) in mg/g was used as the response. The *q_e_* was calculated by the following Equation (1).

(1) $$q_{e}=\frac{(C_{i}-C_{e})V}{m}$$

where *C_i_* and *C_e_* are the initial and the equilibrium concentrations, respectively, in mg/L, *V* is the volume in L and m is the mass of the adsorbent material in grams.

A second-order model was generated according to Equation (2).

(2) $$y=\beta_{o}+\overset{k}{\underset{i=1}{\sum }}\beta_{i}x_{i}+\overset{k}{\underset{i=1}{\sum }}\beta_{ii}x_{i}^{2}+\underset{i<j}{\sum }\sum \beta_{ij}x_{i}x_{j}+\epsilon$$

The adsorption experiments consisted of adding 25 mg of the sorbent material in 25 mL of a solution containing 50 mg/L of Nd^3+^ ions, kept at 120 rpm in an orbital shaker for 24 h prior to the Nd analysis in a microwave plasma–atomic emission spectrometer (MP-AES 4100, Agilent Technologies, Santa Clara, CA, USA). The pH of the solutions was adjusted using convenient solutions of HNO_3_ or NaOH with a concentration of 0.1 M or 0.01 M. 

#### 2.4.2. Kinetic Study

The adsorption kinetics reaction was evaluated by measuring the remaining concentration of Nd^3+^ ions during the time. The experiments consisted of adding 500 mg of BR into 500 mL of a solution which was composed of 100 mg/L of Nd^3+^ and was previously adjusted to pH 4.8. Nonlinear mathematical models including pseudo first-order (PFORE), pseudo second-order (PSORE) and Elovich equations, according to Equations (3)–(5), respectively [23], were fit to the experimental data to evaluate the kinetics of the process. The experiments were carried out in triplicate.

Pseudo first-order rate equation (PFORE):

(3) $$\frac{dq}{dt}=k_{1}\left(q_{e}-q\right)$$

Pseudo second-order rate equation (PSORE):

(4) $$\frac{dq}{{\left(q_{e}-q\right)}^{2}}=k_{2}dt$$

Elovich equation:

(5) $$q=\frac{1}{\beta }ln\left(1+\propto \beta t\right)$$

where *q_e_* and *q* are the equilibrium sorption capacity and the adsorption capacity (mg/g) at any time, respectively, *t* is the time (min), *k*_1_ and *k*_2_ are the PFORE rate constant (1/min) and the PSORE rate constant (g/mg·min), respectively, *α* is the initial adsorption rate (mg/g·min) and *β* is a desorption constant related to the extent of surface coverage and activation energy.

#### 2.4.3. Equilibrium Study

The equilibrium isotherms were obtained by varying the initial Nd^3+^ concentration (*C_i_*) from 10 to 200 mg/L at an initial pH of 4.8. The equilibrium isotherms were modeled by applying the Langmuir, Freundlich and Dubinin–Radushkevich (R-D) nonlinear models, according to Equations (6)–(8), respectively. The experiments were carried out in triplicate.

Langmuir non-linear equation:

(6) $$q_{e}=\frac{q_{max}bC_{e}}{1+bC_{e}}$$

Freundlich non-linear equation:

(7) $$q_{e}=K_{F}C_{e}^{1/n}$$

SIPS non-linear equation:

(8) $$q_{e}=\frac{q_{max}K_{s}C_{e}^{1/ms}}{1+K_{s}C_{e}^{1/ms}}$$

where *q_e_* is the adsorption capacity (mg/g), *C_e_* is the equilibrium concentration (mg/L), *q_max_* is the Langmuir and SIPS adsorption maximum capacity expressed in mg/g, *b* is the Langmuir constant (L/mg), *K_F_* is the Freundlich constant ((mg/g)·(L/mg)^1/n^), *n* is the sorption intensity (dimensionless), *K_s_* is the SIPS equilibrium constant (L/mg) and *ms* is the SIPS model exponent.

#### 2.4.4. Desorption Evaluation

Several adsorption–desorption cycles were carried out using HNO_3_ 0.1 M as the eluent. The recovery of Nd^3+^ was calculated by using Equation (9).

(9) $$Recovery\;\%=\frac{C_{D}*V_{D}}{\left(C_{i}-C_{e}\right)*V_{A}}*100$$

where *C_D_* is the concentration (mg/L) of the desorbed Nd^3+^ ions in the desorption cycle, *C_i_* and *C_e_* are the initial and equilibrium concentrations of Nd^3+^ ions (mg/L) in the adsorption cycle, respectively, and *V_D_* and *V_A_* are the volumes used in the adsorption and elution cycles, respectively. The experiments were carried out in triplicate.

## 3. Results and Discussion

### 3.1. Material Characterization

#### 3.1.1. SEM Observations

**Figure 1:**
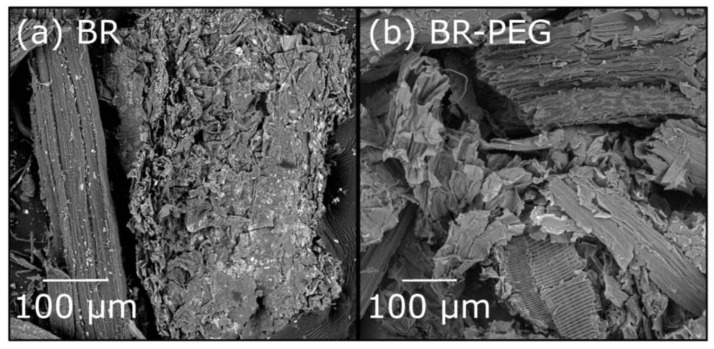
Microscopical observations of materials (magnification: X500). (**a**) neat BR, (**b**) BR–PEG.

The surface morphology observations of BR before and after the sugar production process (BR–PEG) were carried out by SEM (Figure 1). The surface of neat BR presented heterogenous textural properties, combining flower-like rugosity and a smooth surface (Figure 1a). The smooth surface could be attributed to the cementing non-cellulosic layers of the cell wall of BR, including lignin, hemicelluloses and pectin compounds [24]. After the enzymatic hydrolysis with PEG (Figure 1b), the rugosity of the BR–PEG surface partially disappears, and consequently, fibers and smoothness are more appreciable. The fiber-like texture is presented in micro-sized bundles in some parts of the BR–PEG surface. The change in the morphology is caused by the enzymatic hydrolysis previously applied. In addition, the presence of PEG in the BR–PEG aids the dispersity of the fibers. PEG has demonstrated effective effects in minimizing the fiber agglomeration [25], which is also beneficial for the adsorption process because it further increases the available contact area of an adsorbent material.

#### 3.1.2. Compositional Analysis

Neat BR is mainly composed of cellulose (30.66%), followed by hemicelluloses (14.47%) and lignin (12.4%), as depicted in Table 2. As expected, after the enzymatic hydrolysis aided by PEG, some changes in the composition were determined—particularly, the lignin content increased to 62.96%, and the cellulose and hemicellulose concentrations decreased to 17.26% and 10.27%, respectively. The changes in the composition are related to the enzymatic hydrolysis, which consumes the cellulose and hemicelluloses, decreasing their contents but consequently causing an increase in the lignin content. Thus, the material is mainly composed of lignin.

#### 3.1.3. Thermogravimetric Analysis

**Figure 2:**
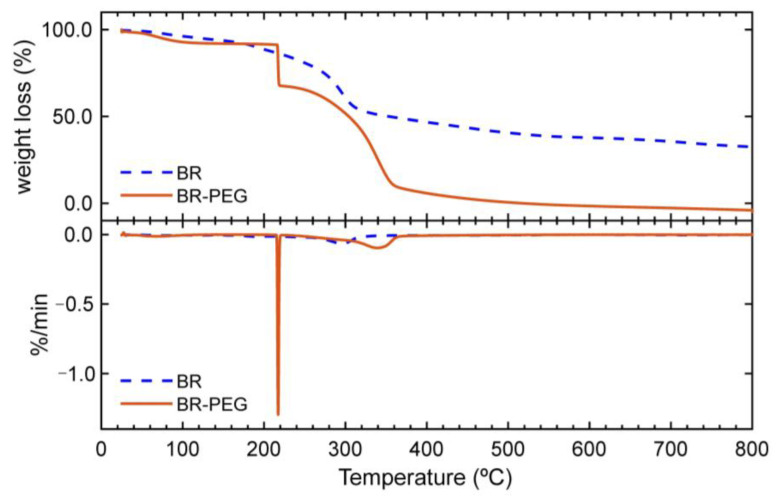
Thermogravimetric profile and DTG curves of neat BR and BR–PEG.

The thermal degradation characteristics provide key information about the stability and composition of a sorbent material, because the thermal behavior depends on the inherent characteristic and molecular interactions of a solid material [24]. The thermogravimetric (TGA) and differential thermogravimetric (DTG) profiles of BR and BR–PEG (Figure 2) showed an initial small weight loss from 35 °C to 120 °C due to the evaporation of the absorbed water of the materials. BR showed an initial degradation from 170 °C to 310 °C, corresponding to the water evaporation and decomposition of extractives, hemicelluloses and lignin [24], and a final weight loss in the range between 300 °C to 380 °C, accounted for by the cellulose decomposition. Two stages of weight loss were observed in the BR–PEG sample: i) an initial and fast decomposition at 217 °C corresponding to the decomposition of PEG and the remaining hemicelluloses and ii) a second significant degradation of ~60% of mass weight from 220 °C to 360 °C due to the thermal degradation of the lignin, cellulose and polyether chain of PEG [26,27].

#### 3.1.4. Infrared Analysis

**Figure 3:**
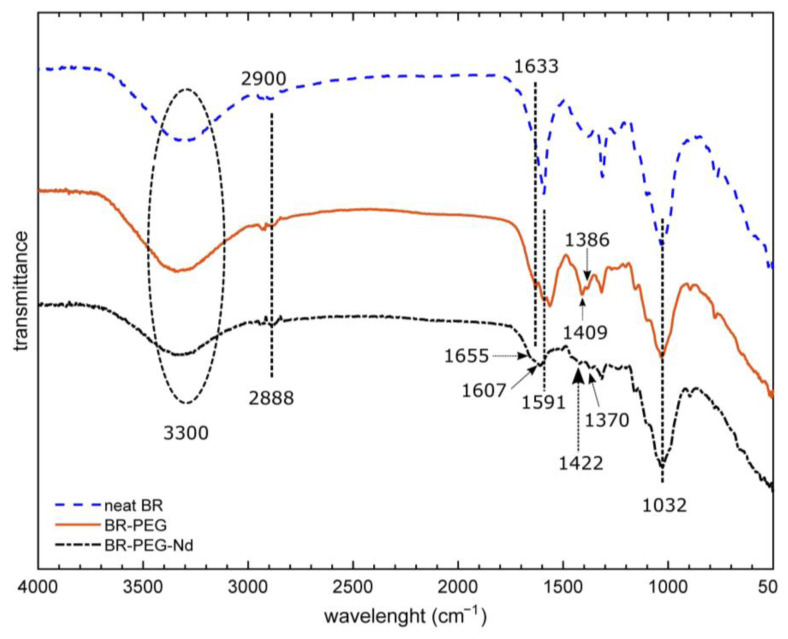
Infrared analysis of neat BR and BR–PEG.

FTIR analysis based on the determination of surface functional groups provided information about the changes in composition of BR, BR–PEG and BR–PEG after contact with Nd^3+^ ions (BR–PEG–Nd) (Figure 3). A broad stretching vibration around 3300 cm^−1^ belonging to the -OH groups is attributed to the lignocellulosic compounds such as hemicelluloses, pectins and cellulose present in the neat BR, BR–PEG and BR–PEG–Nd materials. A change in the stretching vibration from 2900 cm^−1^ in neat BR to 2888 cm^−1^ in BR–PEG is attributed to the hydrogen bonding caused by the incorporation of PEG in the material [28]. Various new peaks in BR–PEG in the fingerprint region between 1700 cm^−1^ and 700 cm^−1^ can be attributed to the lignin compounds [17]. The stretching vibration at 1032 cm^−1^ related to C-O and O-C-O and the aromatic C-H groups related to the hemicelluloses, cellulose and lignin [12,29] remain in BR–PEG as the original neat BR, indicative of the presence of these compounds in both materials. The FTIR analysis indicates that PEG appears incorporated in BR–PEG; both materials maintain the hemicelluloses, cellulose and lignin. These compositional changes revealed by FTIR analysis agree with the chemical and TGA analysis discussed in Section 3.1.2 and Section 3.1.3.

The BR–PEG material presented four shifts in the stretching vibrations after the contact with Nd^3+^ ions during the adsorption process. A bathochromic shift from 1633 cm^−1^ to 1655 cm^−1^ was related to the participation in the adsorption of C=C bands from aromatic rings [17]. The stretching vibration at 1591 cm^−1^, which was attributed to the carboxylic acids of BR [12], changed to 1607 cm^−1^, which is indicative of the interaction of this negative COO^-^ group with Nd^3+^ ions. The peak at 1409 cm^−1^, which was attributed to the methylene vibration of glucopyranosyl [30], experienced a change to 1422 cm^−1^. The aromatic ring vibration, plus the C=O stretching vibration from the lignin, experienced a red change from 1386 cm^−1^ to 1370 cm^−1^. The shifts in these specific stretching vibrations of the BR–PEG surface are related to functional groups originating from the lignin, cellulose and hemicelluloses participating in the adsorption of Nd^3+^ ions.

### 3.2. Adsorption Experiments

#### 3.2.1. pH and Particle Size Factorial Experiments

The pH of the aqueous phase and the particle size of the adsorbent material had been considered as factors influencing the adsorption process [31,32]. This section presents the simultaneous evaluation of two factors such as the pH of the solution and the particle size of the BR–PEG applied to the Nd^3+^ ion recovery. The optimal levels of these two factors were determined to set the conditions prior to performing the equilibrium isotherms, kinetics and desorption evaluation.

The results obtained after CCD experiments were adjusted among four mathematical models including linear, two-factor interaction (2FI), quadratic and cubic models (Table 3). The quadratic model was found to be the best model because the lack-of-fit *p*-value was statistically significant (*p* < 0.05) and the adjusted and predicted R^2^ values showed acceptable values (R^2^ closer to 1), except for the cubic model, which is discarded because the model was aliased. Although the linear and 2FI show statistical significance (*p* < 0.05), the R^2^ (adjusted and predicted) is relatively farther than that shown by the quadratic model. Thus, as expected, the quadratic model was selected for further data analysis.

ANOVA Table (Table 4) shows the main effects and statistically significant interactions of the quadratic model. High F-values together with *p*-values less than 0.05 (95% of the confidence level) define which factor is statistically significant with respect to the model. The quadratic model result is statistically significant based on the high F-value of 207.67 and *p* < 0.0001, defining the accuracy of the model. In addition, the variables *A* (pH) and *A^2^* were significant based on high F-values and *p* < 0.05, while *B^2^* (particle-size) showed a lower effect in the model. On the other hand, the interaction of the variables *B* and *AB* did not show a significant effect on the adsorption phenomena (low F-values and *p* > 0.05). 

**Figure 4:**
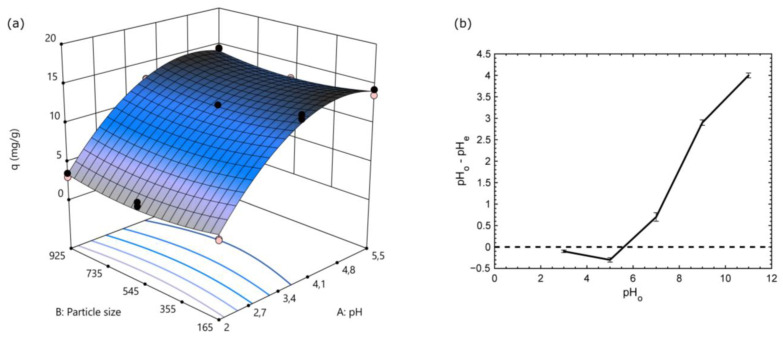
Effect of pH and particle size on Nd^3+^ adsorption capacity. (**a**) surface response and (**b**) pH_pzc_ profile of BR–PEG. (experimental conditions for a): temperature: 25 °C; sorbent dosage, SD: 1 g/L; agitation speed: 180 rpm).

The model result from the quadratic model fitting is represented in Equation (10). Figure 4a shows the surface response.

(10) $$q_{e}=-16.67+13.61*A-0.0084*B+0.0001*A*B-1.4*A^{2}+7.18*B^{2}$$

The main effect influencing *q_e_* was the pH, followed by the pH squared, while the particle size and the interaction *AB* did not significantly affect the response. The optimal levels calculated by the Design Expert 11 software were: pH: 4.5 and particle size: 209.19 µm.

The squared behavior described by pH can be observed in the curvature presented in the pH axis of the modeled surface response depicted in Figure 4a. The lower pH tested (pH 2) presented a lower *q_e_* of around 3 mg/g, while pHs around 4.5 presented around the maximum *q_e_* of 14 mg/g, which is a ca. fourfold increase. The changes in *q_e_* along the pH evaluated can be explained by the variability of the electrostatic forces imposed by the functional groups available in BR–PEG. According to the pH_pzc_ profile (Figure 4b), BR–PEG material is negatively charged between pH 3 and 5.6, and the maximum negativity matches with the optimal pH of 4.8, at which point the maximum Nd^3+^ uptake was achieved. The adsorption is mainly caused by the electrostatic forces imposed by the surface functional groups of the material. In accordance with the FTIR analysis, the surface functional groups are related with carboxylic and carbonyl groups and lignin compounds [12,33]. The carboxylic group becomes anionic (-COO^-^) at pHs < pK_a_ of 2.0 to 5.0. Thus, negatively charged carboxylic groups are the active functional groups responsible for the REE adsorption and corroborate the expected high affinity of rare earths to the oxygenated surface functionalities [34] present in BR–PEG material. 

#### 3.2.2. Kinetics

**Figure 5:**
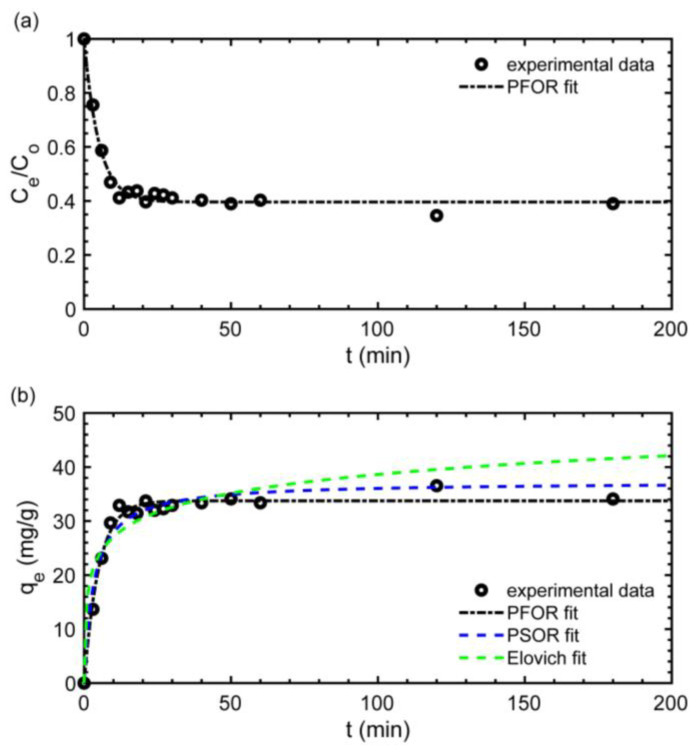
Kinetics modeling of Nd^3+^ ion recovery. (**a**) reaction time vs. concentration, (**b**) reaction time vs. *q_e.._* (experimental conditions: temperature: 25 °C; sorbent dosage, SD: 1 g/L; particle size: 150–180 µm; agitation speed: 180 rpm; pH_o_: 4.5; *C*_o_: 50 mg/L).

The kinetic evaluation provides the information regarding the reaction time for capturing an adsorbate in the solid adsorbent material, the reaction rate at which the adsorption occurs and the probable adsorption mechanisms governing the phenomena [23]. The behavior of the Nd^3+^ ion concentration over the time during the adsorption is shown in Figure 5a, and the mathematical modeling of the kinetic process using three non-linear kinetics model fittings of PFOR, PSOR and Elovich is presented in Figure 5b.

The typical kinetics steps regarding the adsorption stages can be identified in Figure 5a. During the first stage (film diffusion), from 0 to 10 min, the adsorbate is transported speedily from the liquid phase to the primary external surface of the BR–PEG, achieving around 30 mg/g of adsorption capacity. Step 2 (pore or intraparticle diffusion) takes approximately from 10 to 20 min, in which the Nd^3+^ ions are transported from the external BR–PEG surface into the material pores, where experimental adsorption capacities between 31 mg/g and 36 mg/g were reached. Finally, Step 3 (surface reaction) occurs after 20 min until reaching the material saturation. The total reaction time can be 30 min (Figure 5a), which can be considered competitive compared with that of the raw BR material [12] or can be considered fast compared with the natural material chitosan–iron’s reaction time of 5 h [8]. The rapid adsorption is indicative of strong affinities of the material for the adsorbate [35], which corroborates the favorable adsorption determined by the R_L_ factor in Section 3.2.3. Moreover, short reaction times imply a reduced volume reactor during the process scaling and consequently play a key role in reducing cost investments.

Three mathematical models—PFOR, PSOR and Elovich—were fit to the experimental kinetic data (Figure 5b and Table 5). The PFOR model and PSOR presented close values of regression coefficients (R^2^) of 0.98 and 0.96 for PFOR and PSOR, respectively, followed by the Elovich model (R^2^: 0.89). Considering the closeness of the R^2^ values, a statistic error function RMSE (root mean sum-of-squares error) is used to determine which is the best fitting model for describing the kinetic experimental data. The RMSE error function provides a quantification of the error between the model parameter and experimental values and can be used to discriminate when the R^2^ values are too close [23]. Thus, PFOR presents a lower RMSE than the PSOR model (1.38 for PFOR and 1.91 for the PSOR model) and can be considered a model suitable for describing the experimental kinetic data. The PFOR model suggests that the rate of adsorbate occupation is of the first order and is related to the surface sites available, and the adsorption rate is controlled by the diffusion of the adsorbate, while the PSOR and Elovich models are representative of chemisorption [23,36]. Thus, physical sorption is assumed to be the dominant adsorption mechanism to the Nd^3+^ ions on BR–PEG. The PFOR model has been used to describe the adsorption kinetics of a banana peel adsorbing Cd(II) [37] and Cr(VI) [38].

#### 3.2.3. Equilibrium Isotherms

**Figure 6:**
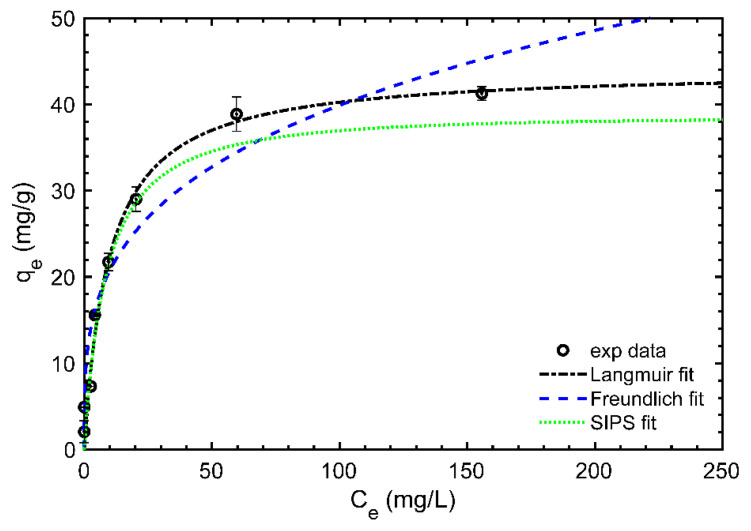
Equilibrium isotherm of Nd^3+^ ion recovery. (Experimental conditions: temperature: 25 °C; sorbent dosage, SD: 1 g/L; particle size: 150–180 µm; agitation speed: 180 rpm; pH_o_: 4.5.)

Equilibrium isotherms represent the capacity of sorbent materials to capture the target element or molecules at different initial concentrations. Figure 6 shows the equilibrium isotherm representation of Nd adsorption on BR–PEG material at different Nd concentrations. The data were fit to three mathematical models: Langmuir, Freundlich and SIPS.

The BR–PEG equilibrium isotherm presents a type I shape (convex upward) [39], showing a deep slope at the beginning until a *q_e_* of 21 mg/g, followed by a horizontal plateau reaching the highest *q_e_* values at around 40 mg/g. Table 6 shows the parameters obtained after fitting the experimental data on the mathematical models evaluated using the Langmuir, Freundlich and SIPS models. 

The Langmuir equation better describes the experimental data (R^2^ = 0.97) among the three non-linear mathematical models applied. Langmuir theory assumes that adsorption occurs in definite localized sites covering a homogeneous adsorbent surface [40]. Therefore, once Nd^3+^ ions occupy a particular surface functional group, those sites cannot be held again. In addition, the R_L_ dimensionless factor indicates a favorable adsorption (0 < R_L_ < 1) [39]. The Langmuir model has been probed as an adequate model for describing equilibrium isotherms where lignocellulosic residues were tested in metal binding, including various parts of banana wastes such as banana rachis for adsorbing critical rare earths [12], banana stalk for removing Pb(II) [41] or banana peel activated carbon for adsorbing Cu(II), Ni(II) and Pb(II) [42]. In addition, BR–PEG showed a better adsorption capacity than other biopolymers such as chitosan, which needs modifications to perform improved adsorption capacities. For instance, chitosan was modified with Fe(OH)_3_, improving its adsorption capacity from 3.77 mg/g to 11.51 mg/g for neat chitosan and chitosan–Fe(OH)_3_ [8].

The *q_max_* calculated by the Langmuir model resulted in 44.1 mg/g, which is compared with similar materials applied in Nd^3+^ ions recovery (Table 7). The BR–PEG results are moderate compared with those of similar materials derived from banana waste including peel (47.0 mg/g) and pseudo stem (66.4 mg/g) and are less than the *q_max_* showed by its neat BR material banana rachis (104.0 mg/g) [12]. In addition, BR–PEG presented a better adsorption capacity compared with other non-modified or natural materials such as bone powder (10.9 mg/g) [43], clinoptilolite (1.8 mg/g) [44], *C. marxianus yeast* (12 mg/g) [45] or chitosan–iron modified material (13.8 mg/g) [8].

Neat BR showed a better adsorption capacity than its residual material BR–PEG. However, the application of BR–PEG represents a route of valorization involving a previous process for producing fermentable sugars and, parallelly, the adsorbent BR–PEG material. This approach provides extra economic profit, because the fermentable sugars obtained during the enzymatic hydrolysis with PEG can be transformed into the most valuable products (i.e., ethanol, ethylene, etc.), representing an advantageous alternative instead of using neat BR directly. In addition, BR in its raw form is able to be used once [12] due to the disintegration of the material, attributed to the solubilization of water-soluble compounds. The reusability of BR–PEG and its advantages compared to neat BR are presented in Section 3.2.4.

In addition, experiments focused on the evaluation of selectivity in solutions containing a mixture of Nd/Fe ions were performed (Figure S1). The initial concentrations simulate a leachate from an Nd/Fe/B magnet, according to the leaching experiments performed by Riaño et al. [46]. The initial concentration of Nd and Fe followed the ratio Fe/Nd: ~6, resulting in the initial concentrations of Fe: 6.69 mmol/L and Nd: 1.10 mmol/L, and prepared in acetate buffer to avoid iron precipitation. The chemical distribution diagrams (Figure S2) show the soluble Fe and Nd ions present in the working solution. The results, in terms of adsorption capacity, indicate no selectivity for Nd ions over Fe ions. Fe ions are preferred 32% more than Nd ions. However, according to the results presented in terms of the yield of extraction (%), the Nd ions were extracted 27% more than the Fe ions, which is because the initial concentration of Fe is higher than that of Nd. BR–PEG material can be used during a concentration stage where the Nd ions, together with some number of Fe ions, can be separated by the adsorbent material, extracting most of the rare earth from the starting solution. That approach is under evaluation, focusing on the selectivity towards rare earths and proposing process strategies towards rare earth extraction and purification. 

#### 3.2.4. Desorption Evaluation

**Figure 7:**
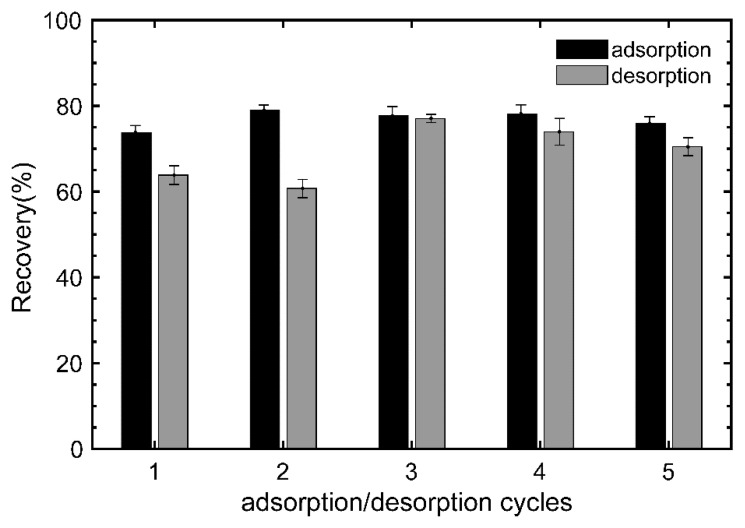
Desorption performance of BR–PEG after several reusages. (Experimental conditions: temperature: 25 °C; sorbent dosage, SD: 1 g/L; particle size: 150–180 µm; agitation speed: 180 rpm; pH_o_: 4.5.)

BR–PEG was reused for five adsorption–desorption cycles to assess its performance of adsorption and desorption using HNO_3_ 0.1 M as the eluent solution (Figure 7). The performance of the adsorption of BR–PEG material remains relatively constant, with over 73% of recovery efficiency throughout the five reusages. The recovery during the desorption showed recoveries >60% during the first two cycles and reached the best recovery in the third cycle, with up to 76%.

BR–PEG presents stability after the adsorption–desorption of Nd^3+^ ions. FTIR spectra (Figure S3) comparing BR–PEG, BR–PEG after Nd^3+^ ions adsorption (BR–PG–Nd) and BR–PEG after the desorption of Nd^3+^ ions (BR–PEG desorbed) were performed to identify changes in the composition after being desorbed with HNO_3_ 0.1 M. It is observed that the main functionalities remain similar after one adsorption–desorption cycle. Stretching vibrations located at 3300 cm^−1^, 1633 cm^−1^, 1409 cm^−1^, 1386 cm^−1^ and 1032 cm^−1^ and related to the OH, C=C, C=O and C-O from lignin, cellulose and hemicellulose did not show changes in their location compared with the starting BR–PEG material, demonstrating the stability in relation to the usage. 

BR–PEG presents stability after the adsorption of Nd^3+^ ions. By comparing the FTIR spectra (Figure 3) between BR–PEG and BR–PG–Nd, it is observed that the main functionalities remain similar after the adsorption (peaks at 3300 cm^−1^, 1316 cm^−1^, 1031 cm^−1^, 1010 cm^−1^ and 1151 cm^−1^) related to the cellulose, hemicellulose and lignin composition of BR–PEG, demonstrating the stability in relation to the usage.

The capacity of an adsorbent material to be reused increases its competitive value against adsorbent materials [47]. BR–PEG material is demonstrated to be a stable material after five reusages. This is to say that, during the whole usage, BR–PEG can adsorb around 200 mg/g (~40 mg/g multiplied by five times) compared with ~100 mg/g for neat BR. The possibility of using BR–PEG several times is a remarkable advantage in relation to neat BR and contributes to the total cost of producing BR–PEG and the circular economy of banana agribusiness. The stability of BR–PEG material is attributed to the presence of PEG in its composition.

## 4. Conclusions

BR–PEG residual material from a saccharification process was probed as an adsorbent material for Nd^3+^ ions recovery from aqueous solutions. BR–PEG showed competitive adsorption capacities and kinetics compared with similar natural-sourced materials. The main mechanisms suggest that adsorption is carried out by electrostatic attraction induced by surface functional groups. The mathematical modeling of equilibrium isotherms suggests that the adsorption was produced in a monolayer. BR–PEG can be considered a sustainable alternative for recovering Nd^3+^ ions due to the fact that this material is valorized from a waste, contributing to the circular economy of the banana market.

## Figures and Tables

**Table 1:** Factors and levels used in CCD experiments.

Factor	Units	Levels		
		−1	0	+1
A: pH	pH	2	3.7	5.5
B: particle size	µm	165.0	550.0	925.0

**Table 2:** Structural composition of the evaluated materials.

Composition	Neat BR	BR–PEG
Hemicelluloses (%)	14.47	10.27
Cellulose (%)	30.66	17.26
Lignin (%)	12.40	62.96

**Table 3:** Fit summary adjusting to different models.

Model	Lack-of-Fit *p*-Value	Adjusted R²	Predicted R²	
Linear	<0.0001	0.7617	0.7097	
2FI	<0.0001	0.7459	0.6836	
Quadratic	0.0025	0.9829	0.9743	Suggested
Cubic	0.0132	0.9902	0.9840	Aliased

**Table 4:** ANOVA Table for the quadratic model.

Source	Sum of Squares	Degree of Freedom	Mean Square	F-Value	*p*-Value	
Model	418.68	5	83.74	207.67	<0.0001	significant
*A*-pH	334.00	1	334.00	828.35	<0.0001	
*B*-particle size	0.0795	1	0.0795	0.1972	0.6643	
*AB*	0.0353	1	0.0353	0.0876	0.7720	
*A*²	82.79	1	82.79	205.33	<0.0001	
*B*²	4.71	1	4.71	11.69	0.0046	
Residual	5.24	13	0.4032			

**Table 5:** Kinetic parameters of BR–PEG.

PFOR				PSOR				Elovich			
*k*_1_ (1/min)	*q_e_* (mg/g)	R^2^	*RMSE*	*k*_2_ (g/mg ∗ min)	*q_e_* (mg/g)	R^2^	*RMSE*	α	β	R^2^	*RMSE*
0.19	33.75	0.98	1.38	0.0079	37.26	0.96	1.91	108.5	0.19	0.89	3.11

**Table 6:** Equilibrium isotherm modeling parametrization.

REE	Langmuir				Freundlich			SIPS			
	*q_max_*	*b*	R^2^	*R_L_* at Ci: 100 mg/L	*K_F_*	*n*	R^2^	*q_ms_*	*K_s_*	*ms*	R^2^
	mg/g	L/mg			(mg^1−1/n^/g L^1/n)^			mg/g	L/g		
Nd	44.11	0.10	0.97	0.0022	10.78	3.52	0.92	44.2	0.10	1.01	0.96

**Table 7:** Nd (III) recovery with different materials.

Sorbent	pH	*q_max_* (mg/g)	Authors
Bone powder	-	10.9	[43]
Kluyveromyces marxianus yeast	1.5	12	[45]
Chitosan/iron (III) hydroxide	6	13.8	[8]
Banana peel waste	4.5	47.03	[12]
Banana pseudo steam waste	4.5	66.46	[12]
Banana rachis waste	4.5	104	[12]
BR–PEG	4.5	44.11	Present work

## Data Availability

Not applicable.

## Supplementary Materials

The following supporting information can be downloaded at: https://www.mdpi.com/article/10.3390/polym15071666/s1, Figure S1: Recovery in a mixture Nd/Fe performed by BR-PEG; Figure S2: Chemical distribution diagram to the Nd/Fe mixture used in the selectivity experiments. (a) Nd ions, (b) Fe ions; Figure S3: FTIR analysis after one cycle of adsorption-desorption.
