# Supplementary material for: Neodymium Recovery from the Aqueous Phase Using a Residual Material from Saccharified Banana-Rachis/Polyethylene-Glycol
Source: Polymers (Basel). 2023 Mar 27;15(7):1666. doi: 10.3390/polym15071666 (PMC10096945; doi:10.3390/polym15071666)
Supplement: Supplementary file 1 [file polymers-15-01666-s001.zip › polymers-2230578-supplementary.pdf]

Supplementary Materials:

Figure S1: Recovery in a mixture Nd/Fe performed by BR-PEG

Figure S2: Chemical distribution diagram to the Nd/Fe mixture used in the selectivity experiments. (a) Nd ions, (b) Fe ions

Figure S3: FTIR analysis after one cycle of adsorption-desorption

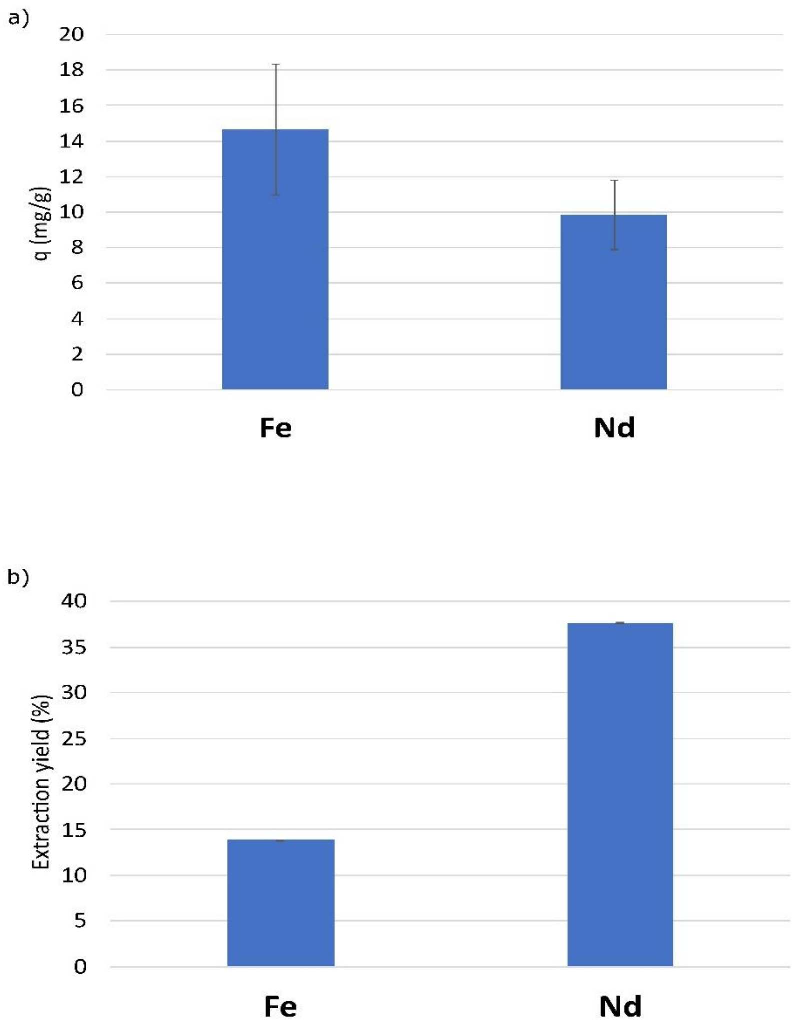

Figure S1. Recovery in a mixture Nd/Fe performed by BR-PEG

(Experimental conditions: initial concentrations: Fe: 6.69 mmol/L, Nd: 1.10 mmol/L; pH_0: 4.5; buffer acetate concentration: 0.5 M acetic acid and 0.01 M sodium acetate; temperature: 25 °C; sorbent dosage, SD: 1 g/L; agitation speed: 180 rpm)

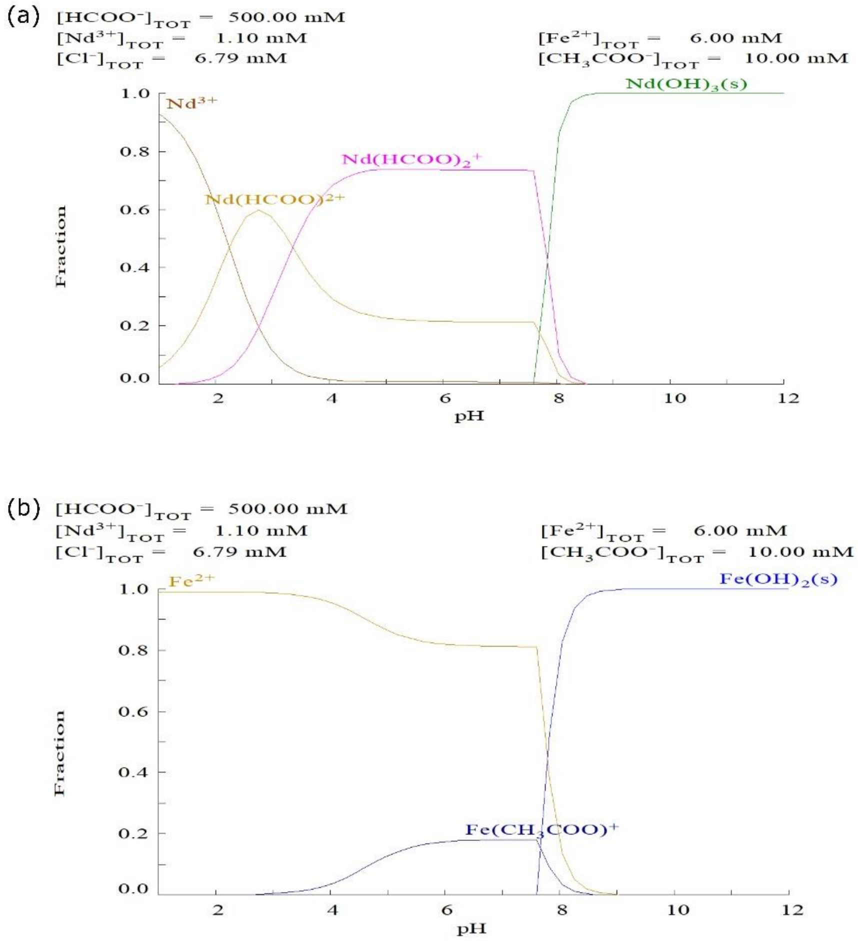

Figure S2. Chemical distribution diagram to the Nd/Fe mixture used in the selectivity experiments. (a) Nd ions, (b) Fe ions

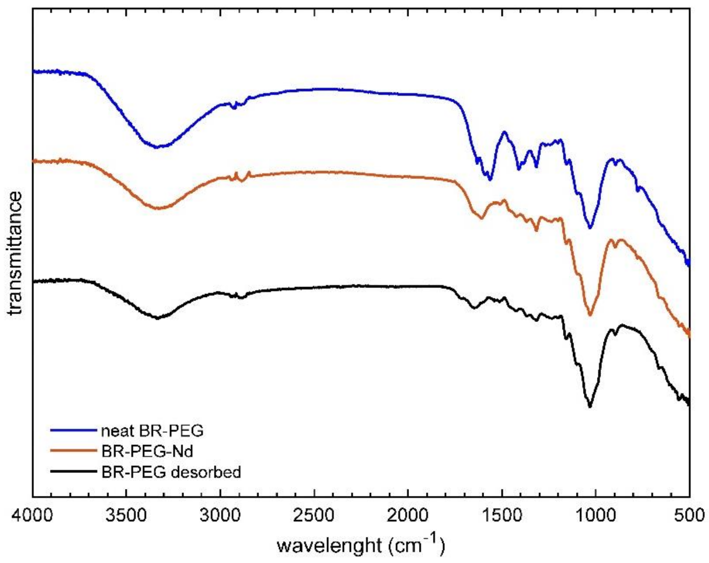

Figure S3. FTIR analysis after one cycle of adsorption-desorption
